# Evaluating Knowledge, Attitudes, and Practices Regarding CalFresh Participation in University Students

**DOI:** 10.3390/nu15010192

**Published:** 2022-12-30

**Authors:** Brittany M. Loofbourrow, Anna M. Jones, Gwen M. Chodur, Suzanna M. Martinez, Leslie C. Kemp, Rachel E. Scherr

**Affiliations:** 1 Department of Nutrition, University of California, 1 Shields Ave, Davis, CA 95616, USA; 2 Aggie Compass Basic Needs Center, University of California, 1 Shields Ave, Davis, CA 95616, USA; 3 Department of Epidemiology and Biostatistics, University of California, 550 16th St., San Francisco, CA 94158, USA; 4 Family, Interiors, Nutrition & Apparel, San Francisco State University, 1600 Holloway Avenue, San Francisco, CA 94132, USA

**Keywords:** food security, college, SNAP, CalFresh

## Abstract

Objective: (1) Identify demographic and academic differences among university students who are food secure or food insecure and (2) explore CalFresh knowledge, attitudes, and practices (KAPs) among university students. Design: A questionnaire, including the 10-item USDA Adult Food Security Survey Module, CalFresh KAPs, and student factors was distributed in Winter 2020 to 10,000 university students. Chi-square test of independence, logistic and linear regressions were used to assess associations between food-secure status and student factors. Exploratory factor analysis determined factors relating to CalFresh KAPs. Kendall’s tau assessed association between CalFresh KAPs factors. Setting: A public research university in California. Participants: Enrolled undergraduate and graduate/professional students (*n* = 10,000). 1535 responses with 1408 included in analysis for having complete data. Results: Food insecurity was associated with: race/ethnicity (Latino/a, OR = 1.97; *p* < 0.001); first-generation status (OR = 2.01; *p* < 0.001); and transfer status (OR = 1.58; *p* = 0.01). Exploratory factor analysis identified five factors related to CalFresh knowledge and attitudes: (1) CalFresh knowledge, (2) Positive attitudes around participating in CalFresh, (3) Negative attitudes around participating in CalFresh, (4) Negative attitudes around others participating in CalFresh, and (5) Fortunate attitudes for not participating in CalFresh. CalFresh knowledge was correlated with positive attitudes towards CalFresh participation (τb = 0.15, *p* = 0.025); negative attitudes towards other individuals’ CalFresh participation (τb = −0.28, *p* < 0.001); feeling fortunate for not needing CalFresh (τb = 0.12, *p* = 0.004); and CalFresh participation OR = 1.40; *p* = 0.02). Conclusions: CalFresh knowledge may influence program participation. Populations who are most impacted by food insecurity should be a focus for improving CalFresh knowledge to promote CalFresh participation.

## 1. Introduction

In the United States (U.S.), food security is defined as access by all people to nutritionally adequate food to support a healthy and active lifestyle [1]. Four levels of food security have been described by the United States Department of Agriculture (U.S.D.A.): (1) high food security, reporting no problems in obtaining food; (2) marginal food security, reporting anxiety regarding food sufficiency or household food shortages; (3) low food security, reporting reduced diet quality, variety, or desirability; and (4) very low food security, reporting disrupted eating patterns and reduced intake [2]. Low and very low food security are described collectively as “food insecure”. In 2020, an estimated 11% of U.S. households experienced food insecurity [1]. Of households experiencing food insecurity, those disproportionately affected included households with non-Hispanic Black (19.1%) and Hispanic (15.6%) members, and households living below 185% of the federal poverty threshold (27.6%), among others [1,3].

College students are another at-risk group for food insecurity who are not highlighted in these national statistics. College students comprise a substantial proportion of the U.S. population, with a projected 19.7 million students attending college during the 2020–2021 academic year [4]. Although college students historically have been considered to be “privileged”, or in an “elite” setting, a significant proportion are from low-income backgrounds [5]. Food insecurity, frequently associated with low income [1], may impact students disproportionately, with the prevalence of food insecurity among college students up to four times higher than the general population [6,7,8,9,10,11,12,13]. Similar to nationwide findings, college students in certain demographic groups are at higher risk, including students who are Black, Hispanic, or from low-income households [6,14]. Food insecurity’s effects may be broad and far-reaching in this group, with negative associations with health [13], psychosocial functioning [15,16], and poor academic outcomes [5,6,8,10,17,18,19,20,21,22]. High academic achievement is of particular concern in this population as the successful completion of college is a driver of social mobility—students who are disadvantaged by the experience of food insecurity may be hampered in their professional growth after leaving their institution due to lower academic achievement [22]. Given that college is often used as a steppingstone in establishing a career path, and that college graduates earn higher wages than non-college graduates, supporting student food security becomes an integral step to their college and future success [22,23].

To reduce food insecurity, the U.S.D.A. established the Supplemental Nutrition Assistance Program (S.N.A.P., previously known as Food Stamps and referred to as CalFresh in California), which provides an average of $155 monthly to its 40 million participants nationwide [24]. College students have been hindered from participating in this program, due to the additional eligibility requirements imposed through the “student eligibility rule”[25] which have limited their participation [26]. In spite of these restrictions many college students are eligible for this program, yet research in this area indicates that their participation is extremely low [10,12,16]. Other reasons for choosing not to participate in programs like SNAP include negative attitudes (such as embarrassment or shame) [27] or lack of awareness of the program or its eligibility requirements, among others [10]. In spite of this, the benefits of participating in SNAP may be manifold, especially in the college population. This resource may help to improve student food security, which research has indicated is associated with improved health and academic outcomes [5,6,7,8,10,13,15,16,17,18,19,20,21,22].

Similar to the U.S., disadvantaged demographic groups across Europe including low-income individuals, women, elderly, single-person and single-parent households, and people with disabilities experience higher food insecurity prevalence [28]. In other wealthy nations, measuring food insecurity is inconsistent, however it also is noted to impact certain groups disproportionately [29]. College students in other high-income nations have also been observed to experience high food insecurity, at proportions that are similar to those in the U.S. [30].

Though CalFresh is helpful in promoting food security and alleviating poverty, its benefits may not be enough to dispel negative perceptions about the program [22,27,31]. In the college student population, reasons regarding low program participation are unknown, however the authors hypothesize that knowledge about food assistance programs is low. Students are often newly independent and may have limited awareness of the many resources available to them including those provided through the university and social programs to improve housing and food access [32,33,34,35]. Alongside the potential benefits of social support programs like CalFresh for students in California and the U.S., the utility of these social food benefits may serve as a model outside of North America, in general and college populations alike [28,36].

Although a growing body of literature continues to illustrate barriers to food security [33,35] and how food insecurity affects college students, a dearth of research shows how student knowledge and perceptions of food assistance resources like CalFresh may differ by food security status [6,7,8,9,10,11,12,13,14,15,16,17,18,19,34]. The purpose of current study was to identify students’ knowledge and attitudes about food access resources, particularly CalFresh, and assess whether knowledge and attitudes were associated with CalFresh participation. In addition, relationships among demographic and academic characteristics, food insecurity, and academic outcomes were assessed.

## 2. Methods

### 2.1. Sample

This was a cross-sectional study conducted during the months of January and February 2020. The campus’s Office of Budget and Institutional Analysis provided a contact list of *n* = 10,000 students representative of the university’s 39,629 students, based on selected factors of race/ethnicity, academic class standing, transfer status, college, international student status, and California residency. Out of this population, *n* = 5000 were generally representative of the university student body. The remaining *n* = 5000 were selected based on the same criteria, with the additional criteria of being recipients of the federal Pell Grant (provided to students from low-income families earning less than $50,000 annually). Of the *n* = 10,000 students contacted, *n* = 1526 students completed the questionnaire (15% response rate). Of these, 100 students were removed for not providing adequate consent to participate. Of the remaining *n* = 1426, *n* = 18 students were excluded for providing incomplete food security data, resulting in an analytical sample of *n* = 1408 participants. Test of differences indicated no demographic differences between students with complete versus incomplete data.

### 2.2. CalFresh Knowledge, Attitudes, and Practices (KAPs) Question Development

Questions relating to knowledge about CalFresh, attitudes regarding CalFresh, campus food access resource and CalFresh participation, and other student lifestyle questions including financial aid receipt and financial habits were developed and edited with the help of a panel of content and survey design experts. Cognitive interviews [37] with a convenience sample of university students (*n* = 15) were conducted to determine whether questions were being answered as intended and to improve clarity. Following edits to refine the questionnaire, a second round of cognitive interviews was conducted (*n* = 10). The final draft of questions was reviewed again by the same expert panel. The questionnaire contained 68 items, with 27 CalFresh KAPs items. Skip logic was implemented in the questionnaire such that not all students viewed all questions. For example, students who indicated current participation in CalFresh also received questions asking about their own participation in the program.

### 2.3. Study Questionnaire and Data Collection

The study questionnaire was administered at the beginning of the January 2020 academic term using a modified Tailored Design Method [38]. At the beginning of the second week of the academic term, potential participants received an initial email invitation to participate, which provided detailed study information, electronic consent letter, and a notification that they would receive a questionnaire via email. A follow-up email was sent one week later with a personalized link, which included informed consent documentation and the questionnaire. The questionnaire was distributed via Qualtrics (Provo, UT, USA) software. In the questionnaire, students electronically consented by providing their university-issued student ID number. Two reminder emails were sent to participants who did not complete the survey, one week apart. Participants who did not complete the questionnaire within the following week received one final reminder. Participants who completed the questionnaire within 3 weeks of receiving the initial questionnaire link were given a $5 gift card incentive.

After data collection via Qualtrics was complete, data were returned to the campus Office of Budget and Institutional Analysis via password-protected electronic file sharing to be combined with student-specific demographic and academic data, including age, race/ethnicity, transfer student status (students transferred from a 2-year or another 4-year institution), low income status (students whose university application indicates a household income below 185% of US federal poverty guidelines), international student status, first-generation status (students whose parents did not complete a 4-year degree), cumulative and term grade point average (GPA), college and major, number of units enrolled, and academic class standing. Combined data were deidentified and returned to the research team for analysis.

### 2.4. Independent Variables

CalFresh KAPs. All participants responded to nine knowledge items including such statements as “My tax dollars help to fund the CalFresh program”, and “CalFresh helps people who are considered low-income”, which were scored using a 3-point Likert scale, including disagree (score = 1), neither agree nor disagree (score = 2), and agree (score = 3) to measure awareness on an increasing ordinal scale. 

Due to U.S. federal protections around student personally identified information and the complexity of CalFresh eligibility criteria [39], participants were asked whether they were CalFresh participants rather than assigned eligibility by the research team. These practices items included questions about their CalFresh participation, whether students currently receive CalFresh benefits or have used them in the past, which were recorded on a yes/no binary scale. Participants who indicated current or past participation in CalFresh received questions regarding their attitudes toward their own and others’ program participation. Participants who indicated no CalFresh participation received questions regarding others’ program participation. 

Attitudes items included 18 total statements, including “I have felt glad”, and “I have felt guilty”, in reference to using CalFresh benefits; “I feel pity for them”, and “I feel glad for them because they are receiving the benefits”, in reference to other individuals using CalFresh benefits; and “I feel fortunate that I don’t need CalFresh benefits” or “I don’t like that I’m not eligible to receive CalFresh benefits” in reference to not receiving CalFresh benefits. All attitudes questions utilized a 5-point Likert scale, ranging from strongly disagree (score = 1) to strongly agree (score = 5). 

### 2.5. Dependent Variables

Food insecurity. Food security status as measured by the 10-item U.S.D.A. Adult Food Security Survey Module (U.S.D.A. A.F.S.S.M.) [40] was self-reported by participants over the previous 30 days. GPA. Cumulative GPA was based on institutional records.

### 2.6. Covariates

The following covariates were included in the regression models: race/ethnicity, first-generation student status, transfer student status, low-income status, international citizenship, out-of-state residency, and academic class standing, including freshman (0–44.99 units accumulated), sophomore (45–89.99 units), junior (90–134.99 units) senior (135+ units) students and graduate/professional students. 

### 2.7. Data Analysis

Descriptive statistics were used to examine demographic and student characteristics. Chi-square analysis of independence was used to compare profile of study sample to university demographics to assess whether study sample was representative of the university population. A Mann–Whitney U test was used to determine if differences in GPA occurred by food security status. 

Exploratory factor analysis was performed with Quartimax rotation to reduce dimensions regarding CalFresh knowledge, general attitudes towards respondents’ own CalFresh participation, attitudes about others’ participation in CalFresh, and attitudes about not needing CalFresh benefits. Kaiser-Meyer-Olkin (KMO) and Bartlett’s test of sphericity were assessed to determine factorizability of KAPs responses. Resulting factor scores were used in Kendall’s tau-b correlation analysis due to the nonparametric nature of variables to determine whether associations existed between knowledge of and attitudes towards CalFresh. All data analyses were performed using IBM SPSS version 27 (Armonk, NY, USA).

Three multiple variable logistic regressions were performed. Model 1 examined transfer student status, first-generation student status, low-income status, race/ethnicity, citizenship, in-state residence, class standing as independent variables and food security status as the dependent variable; Model 2 examined food insecurity, CalFresh knowledge, and the previously listed demographic characteristics as independent variables with CalFresh participation as the dependent variable. Graduate/professional students were used as the reference group, due to their generally higher food security [6].

A third set of multiple linear regression models were used to determine whether food security status was associated with changes in academic performance (GPA). Model 3a included food insecurity as the independent variable; Model 3b included food insecurity, race/ethnicity, transfer status, first-generation status, low-income status, citizenship, California residency, and class standing as independent variables; Model 3c included the same covariates, while omitting graduate/professional students. Considering academic class standing, sophomore students were the reference group in this model—in this class at the university, students are not required to live on campus or to be on a campus meal plan, thus their eating patterns and use of CalFresh may be more representative of other students. Significance for all tests was designated at a *p*-value < 0.05.

### 2.8. Ethical Standards Disclosure

This study was conducted according to the guidelines laid down in the Declaration of Helsinki and all procedures involving research study participants were approved by the University of California Institutional Review Board.

## 3. Results

### 3.1. Sample Characteristics

In the sample, 23% were East Asian (students who identified as Chinese, Korean, or Japanese; Table 1), 28% were Latino/a (students who identified as Chicano, Latino, Mexican, Mexican-American, or Other Spanish), 26% were white (students who identified as white or Caucasian), with other racial ethnic groups having fewer than 10% representation per group. Other demographic characteristics included low-income status (35%), transfer student status (18%), and academic class level (84% undergraduate student and 16% graduate/professional student). Participants provided information on first-generation student status (49%). Chi-square analysis of independence indicated that the race/ethnicity, first-generation, transfer status, and international student status characteristics of the sample were not significantly different from the university population. 

Overall, 43% of respondents had experienced food insecurity (20% low food security, and 23% very low food security. Differences were observed among groups, with students who were identified as Latino/a, a senior student, a first-generation student, a transfer student, being from a low-income background, experiencing disproportionately higher food insecurity. Student financial factors observed to have significant differences in food insecurity prevalence included receiving need-based grants, including the federal Pell Grant, statewide CalGrant (California-specific needs-based grant), and federal work-study. On-campus food access resource use was disproportionately higher among students experiencing food insecurity (Table 2). In addition, students who were food insecure reported higher participation in CalFresh and awareness of CalFresh eligibility.

### 3.2. Exploratory Factor Analysis Findings

Analysis resulted in five constructs of KAPs: (1) *CalFresh Knowledge*, (2) *Positive Attitudes Around Participating in CalFresh*, (3) *Negative Attitudes Around Participating in CalFresh*, (4) *Negative Attitudes Around Others Participating in CalFresh*, and (5) *Fortunate Attitudes for not Participating in CalFresh*. For each, KMO measures and Bartlett’s test of sphericity confirmed that they were likely factorizable. The first analysis presented non-CalFresh participating students with eight statements about CalFresh to assess level of knowledge. One statement was removed due to low communality. With the remaining seven statements, the overall KMO measure was 0.91. Factor analysis identified one component that had an eigenvalue greater than one and explained 49.1% of the total variance. Visual inspection of the scree plot indicated that one component was appropriate to retain—this component was labeled *CalFresh Knowledge*.

The second analysis presented non-CalFresh participants with nine statements about attitudes towards others using CalFresh benefits. The overall KMO measure was 0.8. Factor analysis identified two components that had an eigenvalue greater than 1 and explained 64% of the total variance. Visual inspection of the scree plot indicated that two components were appropriate to retain—these components were labeled *Negative Attitudes Around Others Participating in CalFresh* and *Fortunate Attitudes for not Participating in CalFresh*. 

The final analysis was run on a different subset of the questionnaire, which presented CalFresh participants with 11 statements about attitudes towards using CalFresh benefits. The overall KMO measure was 0.724. Visual inspection of the scree plot indicated that two components were appropriate to retain—these components were labeled *Negative Attitudes Around Participating in CalFresh* and *Positive Attitudes Around Participating in CalFresh.*

Findings from the Kendall’s tau-b correlation examining KAPs factor scores showed that in the relationship between *CalFresh Knowledge and Positive Attitudes Around Participating in CalFresh*, there was a weak positive correlation (τb = 0.15, *p* = 0.025; Table 3). In the correlation between *CalFresh Knowledge* and *Negative Attitudes Around Participating in CalFresh*, there was a moderate negative correlation (τb = −0.28, *p* < 0.001). In the correlation between *CalFresh Knowledge* and *Fortunate Attitudes for not Participating in CalFresh*, there was a weak positive relationship (τb = 0.12, *p* = 0.004). There was not a statistically significant correlation between *CalFresh Knowledge* and *Negative Attitudes Around Participating in CalFresh.*

### 3.3. Food Insecurity

In Model 1, Latino/a students had the highest odds of experiencing food insecurity compared to white students (OR = 1.97; 95% CI, 1.38, 2.83; Table 4); other racial groups did not have a significant difference in the odds of being food insecure. Students who identified as first-generation had double the odds of being food insecure as non-first-generation students (OR = 2.01; 95% CI, 1.52, 2.67), and transfer students had approximately one and a half times the odds of being food insecure (OR = 1.58; 95% CI, 1.12, 2.24) compared to non-transfer students. When sophomore students were considered the reference category of academic class standing, other class standings were not significantly associated with food insecurity. Compared to graduate/professional students, senior students had more than double the odds of experiencing food insecurity (OR = 2.24; 95% CI, 1.43, 3.49). When considered in aggregate, undergraduate students had increased odds of experiencing food insecurity compared to graduate students (OR = 1.48; 95% CI, 1.00, 2.19).

### 3.4. KAPs and CalFresh participation

In Model 2, among a subset of participants who indicated previous or current CalFresh participation (*n* = 437), results showed *CalFresh Knowledge* was positively associated with participation in CalFresh while controlling for demographic and academic factors (Table 5). First-generation and low-income students had higher odds of participating in CalFresh compared to non-first generation and non-low-income students, while freshman and graduate/professional students had lower odds of participating in CalFresh compared to other class levels. Considering undergraduate students collectively compared to graduate students, undergraduate students had nearly five times the odds of participating in CalFresh (OR = 4.57, 95% CI, 1.61, 12.96).

### 3.5. GPA

The distribution of GPA was not similar among the groups, as assessed by visual inspection of GPA distribution. Median GPA for food secure students (3.50) and food insecure students (3.11) was significantly different (U = 166,966, z = −9.22, *p* < 0.001). 

When considered individually and with covariates, food insecurity was associated with lower student GPA (Table 6).

## 4. Discussion

This study sought to identify student knowledge of and attitudes towards CalFresh and assess whether these factors impact CalFresh participation, as well as explore relationships between demographic and academic characteristics, food insecurity, and academic outcomes. Our findings showed that knowledge about CalFresh was correlated with positive attitudes towards the CalFresh program and a higher likelihood of participation in CalFresh. Knowledge about CalFresh was not correlated with negative attitudes towards using the program in CalFresh participants and was correlated with positive attitudes towards other individuals using CalFresh benefits. Food insecurity differed by demographic characteristics, including students from low-income backgrounds, Latino/a and Black/African American students, first-generation and transfer students, and students receiving need-based financial aid. Food insecurity was also found to be negatively correlated with GPA, when considered alone and when controlling for demographic characteristics. 

The food insecurity prevalence results agree with previous work which has shown food insecurity prevalence across the University of California’s campuses to be approximately 42% [12]. Given the size of the University of California (nearly 286,000 of students across 10 campuses), this estimate may represent over 114,000 University of California students who may be experiencing food insecurity, with nearly half of those experiencing disrupted patterns of food intake as indicated by very low food security [12]. Of particular interest to the University of California is the Latino/a population. Nationally, Hispanic individuals make up 18.5% of the population, while in California that proportion is 39.4% [41,42]. At the University of California, Hispanic students make up nearly 25% of the student population [43]; such a large proportion of the university represents an important driver in university metrics, and a considerable number of vulnerable individuals exhibiting a need for improved food security. The current findings indicate that this population is vulnerable to food insecurity and that these students are nearly twice as likely to be food insecure compared to their white counterparts. 

Previous literature has indicated that another important predictor in food insecurity is academic class standing, with one study in a similar population indicating that students who are in the latter half of their university education (particularly juniors and fifth-year seniors) are more likely to experience food insecurity compared to graduate students [6]. The findings of this study expand on those results, indicating that compared to both graduate and freshman students, sophomore, junior, and senior students are more likely to participate in CalFresh, pointing to an increased need for food access once students are likely no longer living on-campus. At the study campus, on-campus housing and meal plans are not required, but over 90% of freshman students do opt to live on campus [44].

Students from low-income backgrounds were observed to have a higher likelihood of experiencing food insecurity, which has been seen in other research [6,20]. The results of this study pointed to a persistent financial struggle related to food security. Students from a low-income background were about 20% more likely to experience food insecurity and receipt of needs-based financial aid (Pell Grant, University needs-based grant) was associated with food insecurity. The consistent association of need-based aid with food insecurity indicates a persistent and pervasive need among low-income students. 

In a 2016 study surveying students across the University of California, food insecurity prevalence was strikingly high (42%), however few students reported using food benefits [6]. Only 2% of students reported participating in CalFresh, a federally funded resource which provides money for food to eligible low-income individuals [12]. One postulation for this low participation was lack of knowledge about the program and its requirements or negative attitudes towards CalFresh. In spite of the myriad of negative consequences associated with food insecurity, the stigma associated with participating in social safety nets which promote food security may discourage participation [27,42].

Despite recent improvements to CalFresh promotion (including legislative efforts to simplify application to CalFresh by college students and improved visibility of the program at the university level) [45], many eligible college students do not participate [10,12]. Previous reports from the UC population have indicated that CalFresh participation is extremely limited [12]. Given the low participation rate, one of the primary objectives of this study was to assess whether stigma associated with CalFresh or uncertainty of eligibility were factors in the lack of participation. This study’s data demonstrates increased utilization compared to 2016 levels [12]. The study population campus had a CalFresh participation rate of 15%, which may indicate a higher level of need at the campus, or that efforts to promote CalFresh (including basic needs center advertisement and hosting a CalFresh representative on campus full-time) have been effective at increasing participation among eligible students. Although participation on the study campus was observed to be higher than previous measurements within the University of California as a whole, overall eligibility knowledge appeared to be lacking. High proportions of students who were food secure and those experiencing food insecurity reported uncertainty of their CalFresh eligibility. In spite of this uncertainty, the population’s attitudes towards CalFresh were overwhelmingly positive. *CalFresh Knowledge* was correlated with positive attitudes towards the program, and inversely correlated with negative attitudes around others’ participation in the program. Additionally, knowledge about the program was correlated with increased program participation. Taken together, these findings suggest that improving program knowledge and understanding of its eligibility criteria may promote student CalFresh enrollment.

This study is the first to examine college students’ knowledge and perceptions of CalFresh/SNAP. Although the overall perceptions of CalFresh appear to be positive, a clear lack of knowledge exists about program eligibility. Previous research in college students indicate that many students, particularly those who are impacted by low and very low food security, would like to receive more information from their institutions regarding food access resources [6]. These results highlight the utility of that notion—increases in knowledge about CalFresh are associated with greater CalFresh participation. Although much has been done in recent years to promote basic needs access on campuses, low knowledge of CalFresh eligibility highlights the continued need for promotion of resources that exist outside of the university campus. Beyond California and the U.S., improving knowledge of available resources to students may not necessarily focus on government subsidized resources, but may instead promote local food banks and community outreach [36]. Indeed, the results of this study point to an increased proportion of students experiencing food insecurity using their local campus-based food resources for support—it logically follows that vulnerable students elsewhere my use resources at a higher prevalence than their peers, and that information campaigns can only serve to bolster participation in such resources [28].

At the time of writing, emerging data from the Census Bureau indicate that cash aid is effective at reducing hardship and alleviating food insufficiency [46]. Taken together, to serve students and other underrepresented populations adequately and promote equity, universities can seek out opportunities to meet the needs of their students and provide them with the resources to help them thrive.

In the college student population, food insecurity has been observed to be detrimental to student physical health, mental health, sleep patterns, and academic outcomes including GPA and retention [6,7,8,10,11,12,13,14,15,16,17,18,19,20]. Educational attainment is an oft-cited way to enhance social mobility and escape the cycle of poverty [23]; students whose abilities are hampered by limited food access may have fewer opportunities to excel in an academic environment, putting them at a disadvantage compared to students who do not experience the same hardships [16,22]. The current study agrees with previous research in this area, describing a detrimental effect of low and very low food security on GPA [6,7,9,14,16,17,18,19,20]. Controlling for demographic factors, food insecurity negatively impacted GPA by 0.12 grade points, which for some students may be enough to depress overall GPA by a letter grade. This lower achievement may preclude them from participating in extracurricular activities or internship opportunities, thus having a farther reach in impact than the immediate student concerns of earning high grades. On a larger scale, these metrics may impact individual students and the institutions they attend. Retention and student GPA impact universities’ standings and perceptions by incoming students. By prioritizing and supporting food access resources like CalFresh, universities can be leaders in promoting equitable access to basic needs while supporting and improving student success. 

Limitations and Future Directions

Limitations of the current study include the cross-sectional nature of the data and self-reporting of CalFresh participation. As these data were collected at one time point, it is impossible to indicate causality of food insecurity and academic performance. Validity and reliability testing of the questionnaire were not performed, as the questions were based on student opinion and were dynamic in nature. Self-reported data may be incomplete or unreliable. State-level differences in SNAP eligibility may have implications for student participation by geography and as such, these findings may not be representative of the reasons for non-participation nationwide. Future research should perform in-depth interviews with students to further elucidate KAPs regarding CalFresh and other food access resources. In addition, students should be followed over time to assess the impacts of food access resources on food insecurity and academic performance.

## 5. Conclusions

The results of this study indicate differences in food secure and food insecure groups in several demographic and academic characteristics among college students, including race/ethnicity, low-income status, transfer and first-generation status, and need-based financial aid receipt. Importantly, these findings indicate that knowledge about CalFresh is associated with positive perceptions of the program and a higher likelihood of participation in CalFresh, pointing to a need for university campuses to expand the reach of advertising for the program. Food insecurity was also found to be negatively correlated with GPA, which offers further support for expanding advertising CalFresh and perhaps other food access resources on campus. Greater visibility of these programs and a clear understanding of eligibility may encourage participation in such programs and reduce food insecurity on campus.

## Figures and Tables

**Table 1:** Demographic and Financial Characteristics of Sample.

	Total	Food Secure	Food Insecure	
	*n* (%)	*n* (%)	*n* (%)	χ^2^ (*p*-Value)
Total Sample (*n* = 1408)	1408	808 (57.4)	600 (42.6)	
**Median GPA** **^†^**	**3.33**	**3.50**	**3.11**	**−9.216 (< 0.001)**
Race/Ethnicity (*n* = 1369)				
American Indian/Alaska Native	11 (0.8)	5 (0.6)	6 (1.0)	0.645 (0.422)
Black/African American	46 (3.4)	23 (2.9)	23 (4.0)	1.061 (0.303)
**East Asian**	**314 (22.9)**	**222 (28.0)**	**92 (15.9)**	**29.295 (<0.001)**
**Latino/a**	**386 (28.2)**	**159 (20.1)**	**227 (39.3)**	**57.033 (<0.001)**
Middle Eastern/South Asian	81 (5.9)	51 (6.4)	30 (5.2)	1.093 (0.296)
Native Hawaiian/Pacific Islander	9 (6.6)	4 (0.5)	5 (0.9)	0.620 (0.431)
Other Asian	43 (3.1)	21 (2.7)	22 (3.8)	1.326 (0.250)
Southeast Asian	130 (9.5)	75 (9.5)	55 (9.5)	0.005 (0.941)
**White/Caucasian**	**349 (25.5)**	**232 (29.3)**	**117 (20.3)**	**15.676 (<0.001)**
**First-Generation Student (*n* = 1239)**	**562 (45.4)**	**277 (38.6)**	**335 (64.2)**	**78.847 (<0.001)**
**Transfer Student (*n* = 1408)**	**253 (18.0)**	**113 (14.0)**	**140 (23.3)**	**20.413 (<0.001)**
**Low-Income (*n* = 1408)**	**491 (34.9)**	**234 (29.0)**	**257 (42.8)**	**29.178 (<0.001)**
**International**	**208 (14.8)**	**133 (16.5)**	**75 (12.5)**	**4.289 (0.038)**
**Out-of-State Resident (*n* = 1408)**	**189 (13.4)**	**125 (15.5)**	**64 (10.7)**	**6.837 (0.009)**
Class Standing (*n* = 1408)				
**Undergraduate Student**	**1190 (84.5)**	**655 (81.1)**	**535 (89.2)**	**17.273 (<0.001)**
Freshman	239 (17.0)	139 (17.2)	100 (16.7)	0.070 (0.791)
Sophomore	240 (17.0)	145 (17.9)	95 (15.8)	1.086 (0.297)
Junior	337 (23.9)	201 (24.9)	136 (22.7)	0.923 (0.337)
**Senior**	**374 (26.6)**	**170 (21.0)**	**204 (34.0)**	**29.649 (<0.001)**
**Graduate or Professional Student**	**218 (15.5)**	**153 (18.9)**	**65 (10.8)**	**17.273 (<0.001)**
**Pell Grant recipient ^‡^ (*n* = 1163)**	**565 (48.6)**	**254 (37.2)**	**311 (64.7)**	**84.858 (<0.001)**
**CalGrant recipient ^‡^ (*n* = 1172)**	**575 (49.1)**	**269 (39.4)**	**306 (62.4)**	**60.389 (<0.001)**
**Subsidized Student Loans ^‡^ (*n* = 1150)**	**376 (32.7)**	**159 (23.7)**	**217 (45.3)**	**59.293 (<0.001)**
**Unsubsidized Student Loans ^‡^ (*n* = 1132)**	**242 (21.4)**	**101 (15.3)**	**141 (29.9)**	**34.757 (<0.001)**
Private Loans ^‡^ (*n* = 1136)	45 (4.0)	26 (3.9)	19 (4.1)	0.028 (0.867)
**University Grant ^‡^ (*n* = 1107)**	**365 (33.0)**	**161 (24.8)**	**204 (44.4)**	**46.698 (<0.001)**
Scholarship ^‡^ (*n* = 1153)	328 (28.4)	204 (30.0)	124 (26.2)	2.069 (0.150)
**Work-Study ^‡^ (*n* = 1127)**	**245 (21.7)**	**112 (17.0)**	**133 (28.4)**	**20.990 (<0.001)**
Own 1 or more Credit Accounts ^‡^ (*n* = 1202)	697 (58.0)	392 (56.2)	305 (60.4)	2.075 (0.150)
**Financial Support from Family or Friend ^‡^ (*n* = 1196)**	**700 (58.5)**	**451 (65.5)**	**249 (49.1)**	**32.147 (<0.001)**
**Have 1 or more unpaid jobs or internships ^‡^ (*n* = 1207)**	**328 (27.2)**	**171 (24.5)**	**157 (30.8)**	**5.990 (0.014)**
**Have 1 or more paid jobs or internships ^‡^ (*n* = 1208)**	**537 (44.5)**	**285 (40.8)**	**252 (49.4)**	**8.787 (0.003)**

† Independent Samples Mann–Whitney U test performed, Z test statistic provided in place of χ^2^. ‡ Data are self-reported.

**Table 2:** Reported Food Access Resource Participation and CalFresh Knowledge and Participation.

	Food Secure	Food Insecure	
	*n* (%)	*n* (%)	χ^2^ (*p*-Value)
No On-Campus Resource Use (*n* = 1306)	392 (51.9)	196 (35.6)	33.821 (<0.001)
Participate in CalFresh (*n* = 1303)	85 (11.3)	128 (76.6)	33.993(<0.001)
Awareness of CalFresh Eligibility (*n* = 1287)			
Yes, and I receive CalFresh	84 (11.2)	129 (24.0)	37.253 (<0.001)
Yes, but I do not receive CalFresh	101 (13.5)	131 (24.4)	25.291 (<0.001)
No, I am not eligible	188 (25.1)	80 (14.9)	19.628 (<0.001)
Not sure	377 (50.3)	197 (36.7)	23.361 (<0.001)

**Table 3:** Kendall’s tau Correlation of CalFresh Knowledge and Attitudes towards CalFresh.

	CalFresh Knowledge Correlation Coefficient (τb)	*p*-Value
Negative Attitudes Around Participating in CalFresh (*n* = 111)	−0.030	0.659
**Positive Attitudes Around Participating in CalFresh (*n* = 111)**	**0.152**	**0.025**
**Negative Attitudes Around Others Participating in CalFresh (*n* = 277)**	**−0.278**	**<0.001**
**Fortunate Attitudes for not Participating in CalFresh (*n* = 277)**	**0.123**	**0.004**

**Table 4:** Regression Model 1: Logistic Regression of Demographic and Academic Characteristics’ Associations with Food Insecurity (*n* = 1206).

Factor	Odds Ratio	CI (95%)	*p*-Value
Ethnicity			
American Indian/Alaska Native	1.486	0.399–5.527	0.555
Black/African American	1.460	0.722–2.952	0.292
East Asian	0.742	0.504–1.091	0.129
**Latino/a**	**1.973**	**1.376–2.828**	**<0.001**
Middle Eastern/South Asian	0.763	0.619–1.923	0.763
Native Hawaiian/Pacific Islander	1.626	0.302–8.758	0.572
Other Asian	1.714	0.857–3.427	0.128
Southeast Asian	1.146	0.710–1.850	0.576
White/Caucasian	Ref	-	-
**First-Generation ^†^: Yes (Ref: No)**	**2.010**	**1.516–2.666**	**<0.001**
**Transfer Status: Yes (Ref: No)**	**1.581**	**1.116–2.239**	**0.010**
Low-Income: Yes (Ref: No)	1.182	0.882–1.585	0.264
Citizen: No (Ref: Yes)	0.927	0.562–1.529	0.766
California Resident: No (Ref: Yes)	1.201	0.705–2.045	0.501
Class Standing			
Freshman	0.886	0.432–1.816	0.740
Sophomore	Ref	-	-
Junior	0.654	0.330–1.297	0.224
Senior	1.234	0.639–2.386	0.531
Graduate or Professional Student	0.527	0.227–1.225	0.137

^†^ Data are self-reported.

**Table 5:** Regression Model 2: Logistic Regression of factors examining KAPs Association with CalFresh Participation (*n* = 437).

Factor	OR	CI (95%)	*p*-Value
**CalFresh Knowledge**	**1.404**	**1.066–1.850**	**0.016**
**Food Insecure**	**2.144**	**1.201–3.827**	**0.010**
Food Secure	Ref	-	-
Race/Ethnicity			
American Indian/Alaska Native	1.267	0.092–17.445	0.860
Black/African American	0.836	0.124–5.658	0.855
East Asian	1.145	0.505–2.599	0.746
Latino/a	1.150	0.526–2.514	0.725
Middle Eastern/South Asian	0.407	0.079–2.088	0.281
Other Asian	0.873	0.205–3.720	0.854
Southeast Asian	0.450	0.139–1.456	0.182
White/Caucasian	Ref	-	-
**First-Generation ^†^: Yes (Ref: No)**	**2.072**	**1.070–4.012**	**0.031**
Transfer Status: Yes (Ref: No)	0.740	0.376–1.455	0.383
**Low-Income: Yes (Ref: No)**	**1.836**	**1.012–3.328**	**0.045**
Citizen: No (Ref: Yes)	0.445	0.103–1.925	0.279
California Resident: No (Ref: Yes)	0.342	0.032–3.614	0.372
Class Standing			
**Freshman**	**0.034**	**0.004–0.277**	**0.002**
Sophomore	Ref	-	-
Junior	1.215	0.538–2.744	0.640
Senior	1.286	0.594–2.785	0.524
**Graduate or Professional Student**	**0.201**	**0.041–0.988**	**0.048**

^†^ Data are self-reported.

**Table 6:** Regression Model 3: Multiple Linear Regression of Food Insecurity’s Association with of GPA.

	Model 3a (*n* = 1381)			Model 3b (*n* = 1187)			Model 3c (*n* = 1019)		
Parameter	B	Std. Error	*p*-Value	B	Std. Error	*p*-Value	B	Std. Error	*p*-Value
Food Insecurity	−0.261	0.0301	<0.001	−0.124	00.307	<0.001	−0.133	0.0348	<0.001
Ethnicity									
American Indian/Alaska Native				0.197	0.1585	0.213	0.338	0.2152	0.116
**Black/African American**				**−0.211**	**0.0859**	**0.014**	**−0.239**	**0.1005**	**0.017**
East Asian				−0.034	0.0434	0.430	−0.031	0.0497	0.528
**Latino/a**				**−0.142**	**0.0432**	**0.001**	**−0.150**	**0.0498**	**0.003**
Middle Eastern/South Asian				−0.098	0.0652	0.131	−0.092	0.0756	0.222
Native Hawaiian/Pacific Islander				−0.086	0.2024	0.673	−0.109	0.2359	0.643
**Other Asian**				**−0.263**	**0.0842**	**0.002**	**−0.299**	**0.0960**	**0.002**
Southeast Asian				−0.016	0.0571	0.778	−0.025	0.0633	0.696
White/Caucasian				Ref	-	-	-	-	-
**First-Generation ^†^: Yes (Ref: No)**				**−0.164**	**0.0339**	**<0.001**	**−0.186**	**0.0388**	**<0.001**
**Transfer Status: Yes (Ref: No)**				**−0.123**	**0.0417**	**0.003**	**−0.123**	**0.0456**	**0.007**
**Low-Income: Yes (Ref: No)**				**−0.075**	**0.0347**	**0.030**	−0.063	0.0374	0.091
Citizen: No (Ref: Yes)				0.012	0.0577	0.831	−0.028	0.0702	0.689
California Resident: No (Ref: Yes)				0.009	0.0606	0.885	−0.001	0.0804	0.986
Class Standing									
**Freshman**				**−0.528**	**0.0550**	**<0.001**	−0.053	0.0522	0.312
**Sophomore**				**−0.427**	**0.0543**	**<0.001**	Ref	-	-
**Junior**				**−0.491**	**0.0520**	**<0.001**	−0.017	0.0496	0.733
**Senior**				**−0.499**	**0.0525**	**<0.001**	−0.022	0.0493	0.652
Graduate or Professional Student				Ref	-	-	—		

^†^ Data are self-reported. Model 3a: Food Insecurity only. Model 3b: All class standings included (Freshman, Sophomore, Junior, Senior, Graduate/Professional Student). Model 3c: Graduate/Professional students omitted. Models include following covariates: Race/Ethnicity, Transfer Status, First-Generation Status, Low-Income Status, Citizenship, California Residency, Class Standing.

## Data Availability

Data sharing not applicable.
